# The transforming acidic coiled coil (TACC1) protein modulates the transcriptional activity of the nuclear receptors TR and RAR

**DOI:** 10.1186/1471-2199-11-3

**Published:** 2010-01-15

**Authors:** Romain Guyot, Séverine Vincent, Julie Bertin, Jacques Samarut, Patrick Ravel-Chapuis

**Affiliations:** 1 Institut de Génomique Fonctionnelle de Lyon (IGFL), Universitéde Lyon, Université Lyon 1, CNRS, INRA, Ecole Normale Supérieure de Lyon, 46 allée d'Italie, 69364 Lyon Cedex 07, France; 2 Hospices Civils de Lyon, Centre Hospitalier Lyon Sud, 69495 Pierre-Bénite cedex, France

## Abstract

**Background:**

The transcriptional activity of Nuclear hormone Receptors (NRs) is regulated by interaction with coactivator or corepressor proteins. Many of these cofactors have been shown to have a misregulated expression or to show a subcellular mislocalization in cancer cell lines or primary tumors. Therefore they can be factors involved in the process of oncogenesis.

**Results:**

We describe a novel NR coregulator, TACC1, which belongs to the Transforming Acidic Coiled Coil (TACC) family. The interaction of TACC1 with Thyroid Hormone Receptors (TR) and several other NRs has been shown in a yeast two-hybrid screen and confirmed by GST pulldown, colocalization and co-immunoprecipitation experiments. TACC1 interacts preferentially with unliganded NRs. In F9 cells, endogenous TACC1 localized in the chromatin-enriched fraction of the nucleus and interacted with Retinoid Acid Receptors (RARα) in the nucleus. TACC1 depletion in the cell led to decreased RARα and TRα ligand-dependent transcriptional activity and to delocalization of TR from the nucleus to the cytoplasm.

**Conclusions:**

From these experimental studies we propose that TACC1 might be a scaffold protein building up a transcriptional complex around the NRs we studied. This function of TACC1 might account for its involvement in several forms of tumour development.

## Background

Nuclear hormone receptors (NRs) constitute a large family, including receptors for retinoids, thyroid (TR) and steroid hormones. They modulate transcription by binding to their respective target along with many regulatory cofactors. NRs repress transcription on positive promoters by binding corepressors, the best known of which are N-CoR and SMRT [1,2]. Histone Deacetylases (HDACs) are recruited and activated in this repressive complex, which then inactivates chromatin, thereby reducing transcription to below basal levels. Ligand binding leads to an exchange between corepressors and coactivators [3,4]. Several proteins bind to this primitive core complex by building interaction networks that eventually lead to activation of the chromatin by histone acetylation. Interactions with basal transcription factors eventually activate RNA polymerase, but similar interactions also play a role during repression.

In mammals, TACC1 belongs, with TACC2 and TACC3, to the Transforming Acidic Coiled-Coil family. TACC proteins share a 200 amino acids C-terminal conserved coiled-coil domain (CC domain), but diverge in their N-terminal part. Many protein variants are derived from each of these three genes [5]. Human TACC genes were initially described as potential cancer genes and are all present in genomic regions that are rearranged in certain cancer cells [6-8], TACC1 having been first discovered as the product of an amplicon in breast cancer [7]. It was shown afterwards that its expression was modified in several cancers [9-15]. The first function for TACC proteins, which was subsequently well documented, lies at the level of the centrosome, where they play a role in stabilizing microtubules during formation of the mitotic spindle at mitosis [16-18]. This function is presumably a factor involved in cell transformation [19]. Other roles for TACC proteins were recognized in the repression of *Xenopus *oocyte translation [20] and in mRNA maturation [9]. A function for TACC proteins in transcriptional regulation was first recognized for TACC3, which was described as an interaction partner and a regulator for a transcription cofactor, ARNT [21]. Since then, several studies have described interactions between a transcription cofactor, a protein belonging to a chromatin remodeling complex or RXRβ and one or several TACC proteins [5,22-25]. Subsequent studies showed that TACC3 can control hematopoeitic cell differentiation through interaction with the transcription coregulator FOG-1 [26] and can relieve transcriptional repression on methylated promoters via an association with the methyl binding protein MBD2 [27]. This raises the possibility that the aberrant expression of one or more TACC proteins may affect gene regulation through their interaction with transcriptional cofactors and components of chromatin remodeling complexes, thus contributing to the oncogenic processes described above.

In this study, we looked for the putative protein partners of TRα through a yeast two-hybrid assay. We identified TACC1 as one such partner and also found that TACC1 is able to bind to several unliganded NRs. Through a functional assay using siRNA to down-regulate TACC1 in animal cells we concluded that TACC1 participates in the overall regulatory function of TR and RAR on target gene expression. Moreover, we demonstrated that TACC1 can control TR and RAR subcellular localization. Our findings provide, for the first time, a general role for TACC1 as a cofactor in the transcriptional regulation of several NRs.

## Results

**Figure 1:**
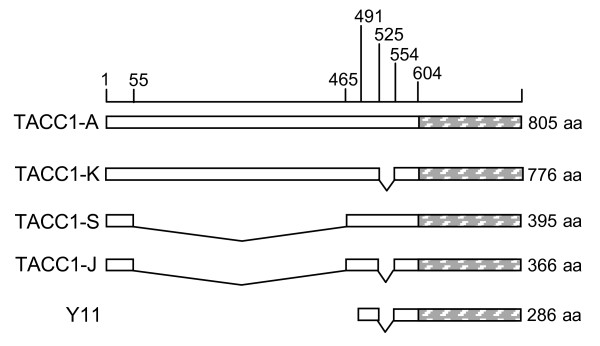
**Representation of hTACC1 isoforms (A, J, K, S and the peptide Y11)**. Numbering represents the amino acid positions on the seminal human TACC1-A protein [7]. Each number points to the first amino acid of the following domain. The hatched region is the coiled-coil domain (202 Amino Acids (AA), from AA 604 to AA 805). For the nomenclature and schemes of the different isoforms, see [9,13]. TACC1-K, -S and J proteins are natural variants. Compared to TACC1-A, TACC1-K protein is a novel isoform that lacks the exon 5 C-terminal domain; exon 5 in the mature mRNA is 121 bases long (see the sequence in TACC1-G, Genbank accession # BC041391). This short exon 5 appeared erroneously as a short exon 6 in TACC1-G and -J on Figure 6 in Lauffart *et al*. [31] (Ivan Still, pers. comm., with permission). The TACC1-S protein lacks domains corresponding to exons 2 and 3. The TACC1-J protein lacks the exon 2 and 3 domains and has the short exon 5 domain [31]. We found all four TACC1's in activated human RAJI cells; we found only TACC1-K and -J in mouse cells. TACC1-Y11 is the peptide found to interact with TRα2 in the yeast two-hybrid screen. Its mRNA contained the coding sequence of the C-terminal domain of the TACC1 protein, plus part of the 3'UTR sequence. The peptide begins at AA 491 and ends at AA 805. So the Y11 peptide (286 AA) corresponds mainly to the TACC1 coiled-coil domain. AA 525 to 553, corresponding to the C-terminal part of exon 5, are missing in Y11.

A mouse full-length TRα2 cDNA was used as a bait to screen a mouse cDNA library. Twenty five clones showing interaction with TRα2 were obtained. Sequencing revealed that they belong to eight different cDNA families. Six clones represented three different cDNAs encoding different lengths of the C-terminal region of the protein N-CoR. This result demonstrated that TRα2 interacts with the NR corepressor in yeast whereas we (data not shown) and others [28] observed that this was not the case in mammalian cells. One cDNA that gave an interaction was the clone Y11, which encoded part of the TACC1 protein (Fig 1).

### TACC1 interacts with several Nuclear Receptors

**Figure 2:**
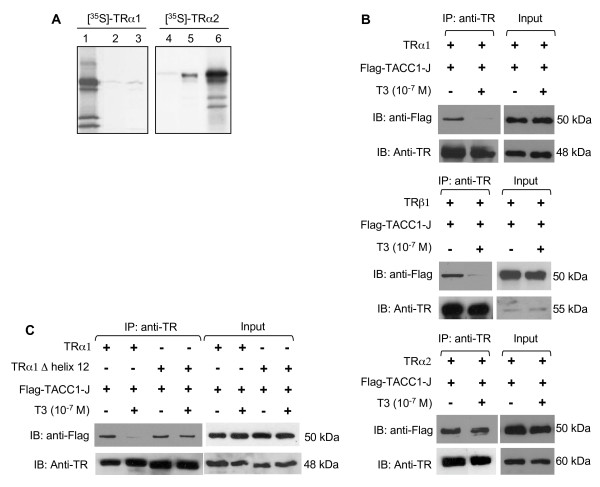
**Interaction of TACC1 and TRs *in vitro*, in yeast and in mammalian cells**. (A) GST-pulldown assays were performed using *in vitro *translated [^35^S]-TRα1 (left) or [^35^S]-TRα2 (right) incubated with GST-glutathione-Sepharose (lanes 2 and 4), or GST-hTACC1-J-glutathione-Sepharose (lanes 3 and 5). Lanes 1 and 6 are [^35^S]-TRα1 or [^35^S]-TRα2 inputs (12.5%). (B, C) Flag-TACC1-J expression plasmid was cotransfected into COS-7 cells with TRα1, TRα2, TRβ1 (B) or TRα1 deleted from Helix12 expression plasmids (C), in the absence or presence of thyroid hormone [T3 (10^-7^M)]. Forty-eight hours after transfection, whole cell lysates were prepared and immunoprecipitated with anti TR antibody. Immunoprecipitates were resolved by SDS-PAGE and blotted with anti-flag and anti-TR antibodies. Inputs correspond to 1% of the proteins used for the coimmunoprecipitation assay.

We observed in a GST-pulldown assay that TRα2 significantly interacted with the shortest human TACC1-J isoform, whereas interaction with TRα1 appeared much weaker (Fig. 2A).

**Figure 3:**
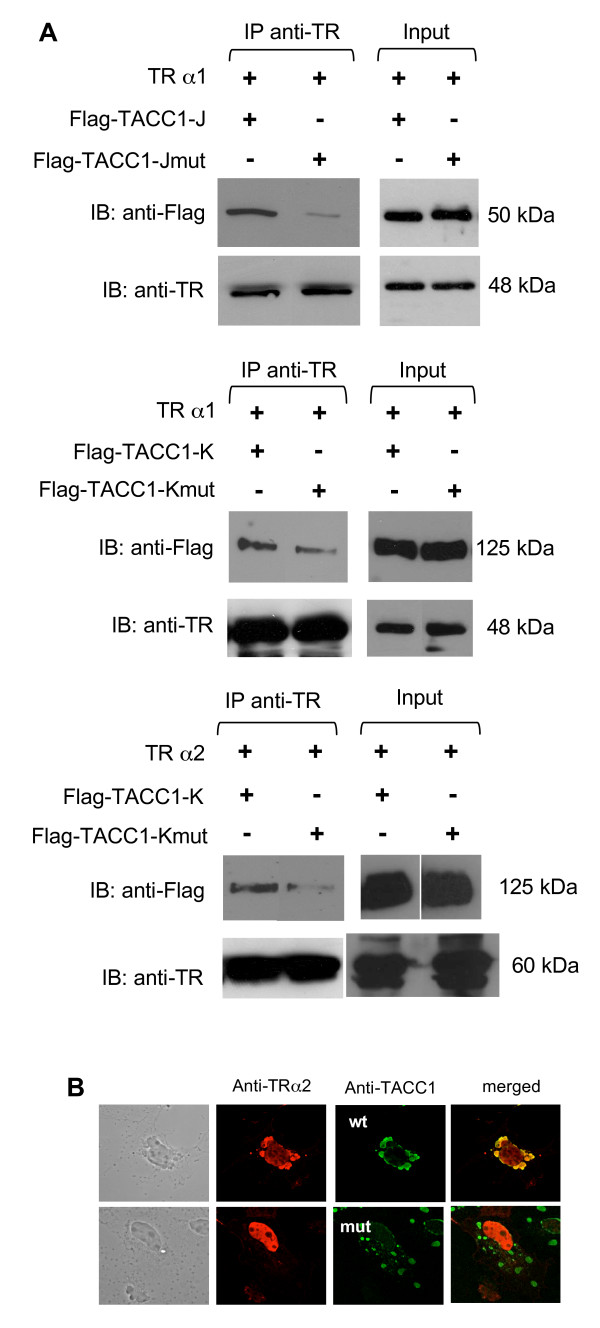
**Interaction of TACC1 or mutant TACC1 and TR in mammalian cells**. (A) Flag-TACC1-J, Flag-TACC1-Jmut, Flag-TACC1-K or Flag-TACC1-Kmut (mut refering to mutations L609A and I610A) expression plasmids were cotransfected with TRα1 or TRα2 expression plasmids into COS-7 cells in the absence of T3 hormone. Forty-eight hours after transfection, whole cell lysates were prepared and immunoprecipitated with anti-TR antibody. Immunoprecipitates were resolved by SDS-PAGE and blotted with anti-flag and anti-TR antibodies. Inputs correspond to 1% of the proteins used for the coimmunoprecipitation assay. (B) Confocal microscopy analysis of the localization of co-transfected TRα2 and TACC1-K (wt) or TACC1-K mutant (mut) in COS-7 cells.

We observed that this interaction between the natural TACC1-J protein and both TRα2 and TRα1 isoforms also occurred in a yeast two-hybrid assay (see Additional file 1). In these cells the interaction between TACC1 and TRα1 decreased when treated with T3. To check for interactions between TACC1 products and TR isoforms in mammalian cells, we performed co-immunoprecipitation assays in COS-7 cells co-transfected with either TACC1-K or -J cDNAs and either TRα1, TRα2 or TRβ1 cDNAs. Clear interactions between TACC1-K and -J proteins and TR proteins were revealed (Fig 2B and 3A). The presence of the ligand T3 significantly decreased the interaction with TRα1 and TRβ1 but, as expected, had no effect on the interaction with TRα2 (Fig 2B). Helix 12 is known to participate in corepressor dismissal when T3 binds to TRα1 [29]. Indeed helix 12 deletion impeded T3 from releasing TACC1-J from TRα1 (Fig 2C). Further experiments indicate that TACC1 amino acids (AA) 609 (Leu) and 610 (Ile) (hTACC1 full-length protein numbering) were essential for interaction with TRα1 and TRα2 in yeast (data not shown). They also appeared to determine strongly or weakly the interaction in mammalian cells for both TRs (Fig 3A).

To further confirm that TACC and the TR proteins can interact in a cellular environment we investigated the localization of these products when expressed in transfected COS-7. Cotransfection of TRα2 and TACC1-J wild type (WT) or mutant (mut) showed that part of TRα2 colocalized with wild type TACC1 (Fig 3B). The mutation of AA 609 and 610 abolished the colocalization. Similar results were obtained with HeLa cells (data not shown).

Taken together, these results demonstrate that unliganded TR proteins and TACC1 do interact *in vitro *and in mammalian cells.

We tested interactions of TACC1-J through co-immunoprecipitation assays in COS-7 cells with several nuclear receptors including RARα, RXRα, PPARγ, GR, and ERα in the absence or presence of their respective ligands (see Additional file 2). All tested receptors showed a clear interaction with TACC1 only in the absence of their ligand. The reduction in interaction was dependent on the dose of ligand as shown for RXR and GR (see Additional file 2). Overexpressed GFP-TACC1-A proteins displaced endogenous nuclear RARα (see Additional file 3).

These data suggest that interaction with TACC1 in mammalian cells is a shared property of several unliganded nuclear receptors of both the steroid receptor and TR families.

### Endogenous TACC1 interacts with RARα in the nucleus and is present in a nuclear chromatin-enriched fraction

**Figure 4:**
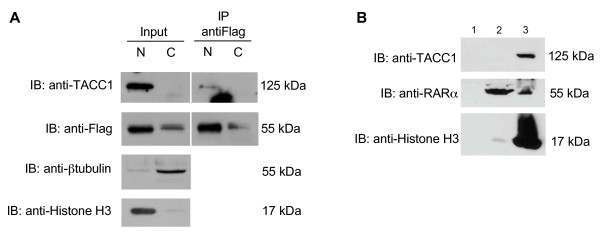
**TACC1 interacts with RARα in the nucleus of F9 cells and is present in a nuclear chromatin-enriched fraction**. (A) Cells from an F9 cell line stably expressing Flag-RARα were fractionated into cytoplasmic (C) and nuclear (N) fractions. 800 μg of total protein of each fraction were immunoprecipitated with an anti-Flag antibody. Immunoprecipitates were resolved by SDS-PAGE and blotted with anti-Flag or anti-TACC1antibodies, which reveal only the longest TACC1 proteins (A and K). Absence of cross contamination between cytosolic and nuclear fractions was verified using respectively anti-β-tubulin and anti-Histone H3 antibodies. The same amount of protein was loaded in each lane. (B) F9 cells were fractionated into cytosolic (lane 1), soluble nuclear (lane 2) and chromatin-enriched (lane 3) fractions. Cell-equivalent amounts of fractions were probed by immunoblotting with anti-TACC1, anti-RARα, and anti-Histone H3 antibodies.

We found that F9 embryonic carcinoma cells expressed fairly high levels of endogenous TACC1. They appeared to be the best cellular model to analyse in more detail the intracellular localization of TACC1 and its interaction with nuclear receptors. Cell fractionation revealed a strong association between TACC1 and the nuclear pellet (Fig 4A). The nuclear interaction of TACC1 with a nuclear receptor was thus explored in co-immunoprecipitation assays in F9 cells stably expressing a recombinant Flag-RARα. In these cells, Flag-RARα protein overexpression is only 5 fold compared to endogenous RARα proteins (data not shown). The relative amounts of Flag-RARα and TACC1 in the supernatant versus the nuclear fraction were determined. As illustrated in Figure 4A, most TACC1 and RARα proteins were found in the nuclear fraction of cell extracts. In the co-immunoprecipitation assays we observed that immunoprecipitation of the nuclear Flag-RARα brought down the endogenous TACC1 protein. We conclude from these results that, in F9 cells, endogenous TACC1 does interact with RARα within the nucleus.

The interaction of TACC1 with nuclear receptors within the nucleus of the cell led us to speculate that TACC1 might be a chromatin protein. To explore this further we prepared cytosolic, soluble nuclear and chromatin-enriched fractions from F9 cells using a sequential high salt extraction procedure. We observed TACC1 to be localized in the chromatin-enriched fraction (Fig 4B). To test the quality of the extraction procedure, we checked the distribution of histone H3 and RARα proteins in the two fractions (Fig 4B). As predicted, histone H3 was mainly found in the chromatin-enriched fraction and RARα in both fractions. These results suggest that TACC1-RARα interactions occur at the level of the chromatin in the nuclear compartment.

### TACC1 enhances Transcriptional activation by TRα and RARα

We first tried to determine the function of TACC1 in a functional assay. Interaction was assayed with a full-length TRα1 and a CAT reporter gene under the control of a thyroid hormone response element, DR4, in HeLa cells. Neither VP16 nor TACC1-Y11/VP16 alone activated the reporter (data not shown). TRα1 alone activated transcription in the presence of the hormone, as predicted. Co-expression of the fusion protein TACC1-Y11/VP16 repeatedly over-activated transcription by 2.5 to 7 fold in many experiments, an effect dependent on T3 (data not shown). These results demonstrate that a functional interaction does occur with TRα1 on the target DR4 in the presence of T3. The presence of VP16 in the fusion protein prevented us from reaching a conclusion on the function of Y11. So we tried to investigate the role of TACC1 in regulating gene expression with TACC1 proteins devoid of VP16. However, although we designed many TACC1 variants, every protein mostly stayed in the cytoplasm when it was overexpressed.

**Figure 5:**
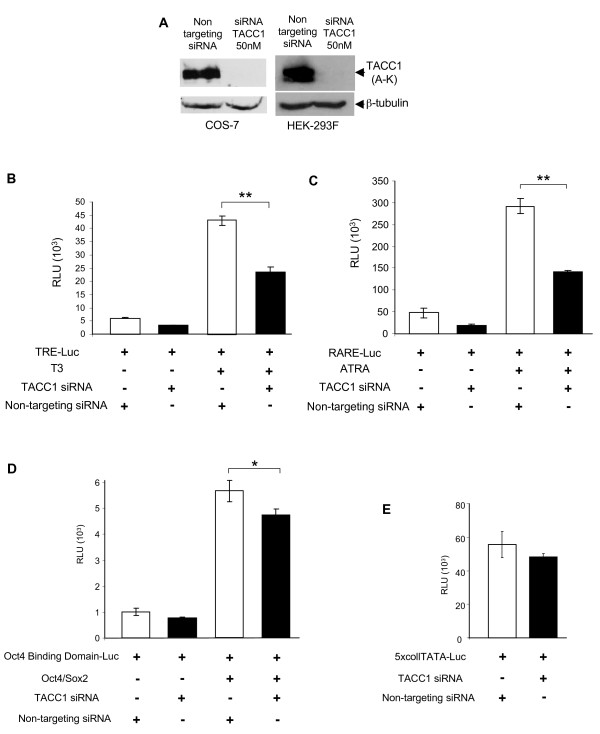
**TACC1 participates to the modulation of transcriptional activity by nuclear receptors**. (A) The protein levels of TACC1 in control (non-targeting siRNA) or TACC1 siRNA treated cells were determined by immunoblotting with an anti-TACC1 antibody (Upstate). β-tubulin was used as a loading control. (B, C, D, E) Non-targeting siRNA or TACC1 siRNA treated HEK-293F cells were transiently transfected with TRE-TKLuc and TRα1 cDNA expression vector (B), RARE-TKLuc (C), Oct4-Binding Domain-Luc and Oct4 and Sox2 expression plasmids (D) or 5xcoll TATA Luc (E). Cells were treated with 10^-7^M of T3 (B) or all-trans RA (ATRA) (C) for 48 h. Firefly luciferase was normalized for cotransfected Renilla luciferase activity (RLU). Open histogram, control siRNA treated cells; black histogram, TACC1 siRNA treated cells. The *bars *indicate the mean ± S.E. of triplicate transfections from a representative experiment (* p < 0.05, ** p < 0.01, by Student's-test).

We thus used an alternative strategy aimed at knocking down endogenous expression of TACC1 in cells in culture through RNA interference using a mixture of four siRNAs designed from the human TACC1-J sequences, in such a way that every TACC1-A, -K, -S, -J mRNA and most of the other TACC1 mRNAs depicted in Genbank and in the literature [30,31] should be targeted. The siRNAs against human TACC1 were transfected into HEK-293F cells or simian COS-7 cells. We verified that they were completely or severely depleted in the mRNAs for TACC1 but not for TACC2 and TACC3 mRNA (see Additional file 4). Long TACC1 proteins (A and K) were totally absent (Fig 5A). It was not possible to test the absence of the shortest proteins (S and J) with the antibody used.

We then tested, in the treated HEK-293F cells, the response of a reporter gene to activation by T3. The cells were co-transfected with a TR-expression plasmid and a luciferase reporter construct driven by a chicken E6 lysozyme Thyroid hormone Responsive Element (E6-TRELuc). In control HEK-293F cells treated with a non-targeting siRNA, reporter expression was activated by T3. In contrast, in TACC1 siRNA treated HEK-293F cells, activation by T3 was decreased by 46% (Fig 5B). In COS-7 cells activation by T3 was even more severely decreased (data not shown). We also analysed in HEK-293F cells the transcriptional activity of endogenous RAR in response to all-trans retinoic acid (ATRA) on a transfected luciferase reporter construct driven by a Retinoic Acid Response Element (RARE). Activation of the reporter expression was decreased by 50% in cells treated with TACC1 siRNAs (Fig 5C). As controls we checked the effects of siRNA on transcription induced by the Oct4 (Fig 5D) and AP1 (Fig 5E) transcription factors. As shown in Figs. 5D and 5E, down-regulating TACC1 in the HEK-293F cells affected transactivation by these factors to a less extent. Therefore the effects of TACC1 siRNA were rather more specific to transcription by nuclear receptors.

**Figure 6:**
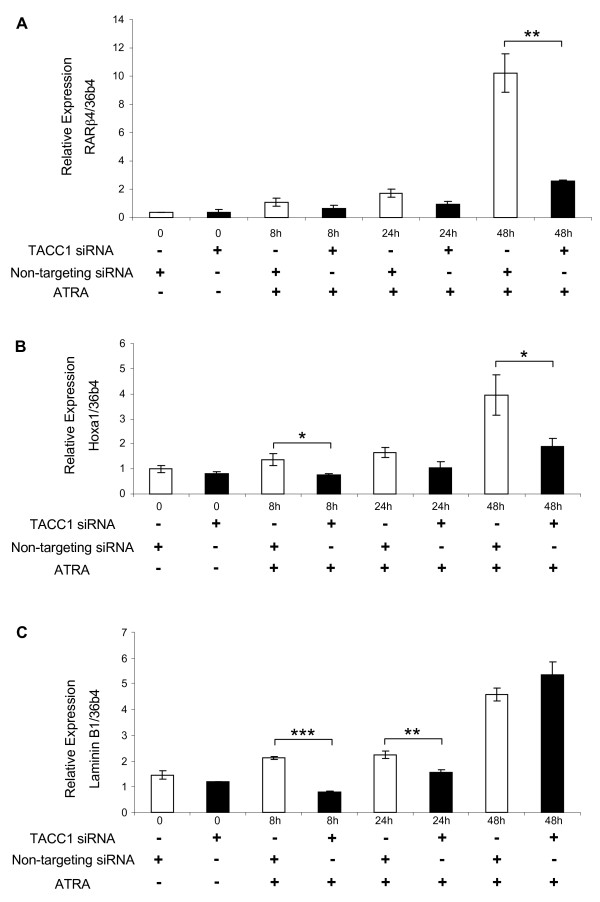
**TACC1 contributes to all-trans RA activation of RAR target genes**. Induction by ATRA of three RAR target genes [RARβ4 (A); Hoxa1 (B); Laminin B1 (C)] in non-targeting siRNA (open histogram) and TACC1 siRNA (black histogram) treated COS7 cells is measured by quantitative RT-PCR. Cells were treated with siRNA for 48 h before being treated with ATRA (10^-7 ^M) for 48 h and the RNAs were extracted at different time points. The constitutive gene 36b4 was used as a control. The error bars represent the SEM (* p = 0.08, ** p < 0.05, *** p < 0.01, by Student's-test).

To further investigate the role of TACC1 on the expression of endogenous genes under more physiological conditions, we analysed the regulation of expression of endogenous retinoic acid-target genes in COS-7 cells. The genes laminin B1, RARβ and Hoxa1 are known to be directly regulated by all-trans RA through RAR [32-34]. We monitored their expression for 48 hours following ATRA stimulation, after two days of siRNA treatment. They showed a reduced activation by ATRA in TACC1-deprived cells, the extent of reduction being dependent on the gene and the time of the kinetics (Fig 6).

We checked that in the conditions of experiments depicted on Figures 5 and 6 the levels of TR and RAR mRNAs were not decreased (data not shown). This suggests that these observations are a direct effect of the TACC1 knockdown.

Taken together, these data show that decreases in the level of expression of TACC1 closely correlate with the decrease in regulating activity of TR and RAR on natural target genes and thereby demonstrate that TACC1 is directly involved in the control of gene expression by several NRs.

### Interaction with TACC1 changes the intracellular localizations of TR

**Figure 7:**
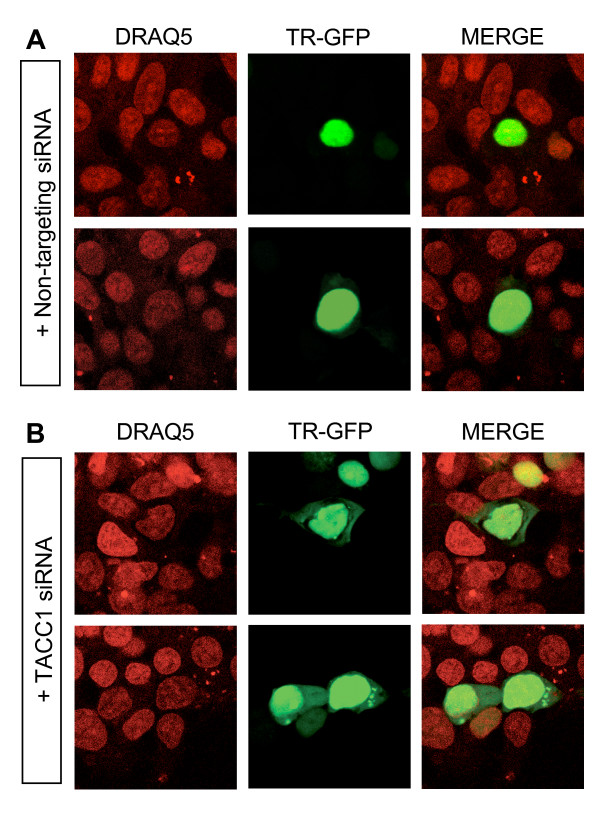
**Interaction with TACC1 changes TR intracellular localization**. The GFP-TRα1 construct was transfected into non-targeting siRNA (A) or TACC1 siRNA (B) treated HEK-293F cells. The distribution of GFP-TRα1 was analysed by confocal microscopy. The cell nuclei were stained with DRAQ5 (red colour). Two representative pictures are shown for each condition. We never observed the GFP fluorescence in the cytoplasm of the control cells.

To further address the question of how TACC1 acts on TR function, we analysed whether the suppression of endogenous TACC1 protein by transfecting HEK-293F cells with siRNAs would alter the intracellular distribution of TR. We analysed the distribution of the fusion protein made from a GFP-TRα1 construct in control and TACC1 siRNA treated cells. As shown in Fig 7A, in control cells the GFP-TRα1 signal was localized exclusively within the nucleus independently of the intensity of the fluorescence. In contrast, in TACC1-deprived cells, the GFP-TRα1 protein was partly located in the perinuclear cytoplasm in a significant proportion of the TR-expressing cells (Fig 7B). The TACC1 protein might therefore be involved in TR mobilisation within the nucleus. Some results suggest that it would be the same for endogenous RARα proteins.

## Discussion

The TACC1 protein and other members of the TACC protein family are known to be associated with the centrosomes and the mitotic spindle during mitosis [10,16,35]. Other studies have shown that TACC proteins are involved in mRNA translation (TACC3 or Maskin) and could be associated with mRNA maturation. We show in this study that TACC1 also plays a role in the control of transcription by nuclear hormone receptors and in the control of their nuclear localization. TACC1 physically interacts with TRα1, TRα2 and TRβ1 in yeast and mammalian cells as shown by several experimental approaches. TACC1 also interacts with RXRα, RARα, PPARγ, ERα and GR (and AR, preliminary results, not shown), transcription factors belonging to the two nuclear receptor families. These interactions involve only aporeceptors as ligand binding abolishes the interaction. In the light of these results and other reported interactions with transcription cofactors, it would appear that TACC proteins play an important role in transcriptional regulation.

### Role of TACC1 in transcriptional control

Our present data clearly show that part if not all the TACC1 protein within the nucleus is associated with chromatin and that endogenous TACC1 interacts with TR and RAR presumably within this chromatin bound fraction.

A putative role for TACC1 in controlling transcription by TR and RAR is suggested by the effects of knocking down protein expression using siRNAs. In cells deprived of TACC1 the ligand induced transcriptional activation of TR and RAR target genes was strongly decreased. This effect was observed on a transfected reporter gene, as well as on endogenous cellular genes. This observation leads to the suggestion that TACC1 works as a coactivator. Similar conclusions were drawn for TACC2 which interacts with RXRβ, on the lactoferrin promoter [25].

Therefore TACC1 is necessary for the overall regulatory activity of TRs, RARα (our results), RXRβ [25] and presumably the other NRs we studied on target gene expression, but its function might be complex. What might be the mechanism of action of TACC1 as regards nuclear receptors? It has been shown that TACC proteins are able to physically interact with coactivators GCN5, pCAF and CBP/p300, and with the chromatin remodelers and regulators GAS41, INI-1 and MBD2. TACC3 can be recruited by the latter on methylated promoters and TACC3/MBD2 may form a complex with HAT and reactivate transcription from methylated genes [22,23,27]. GCN5 also binds to TR [36]. To explain how TACC1 could act as a coactivator in the presence of T3, despite the fact it no longer interacts with TR, a coactivator (such as GCN5) could be proposed as a bridge between TACC1 and TRs, permitting a functional interaction without physical interaction. This can explain why we observed a functional interaction in the presence of T3, as demonstrated by the effect of TACC1-Y11/VP16 on TRα1 on a TRE. A similar behaviour has been described for other transcription cofactors such as Rap46 and PGC-2 [37]. After coactivator binding on TACC1, this protein could participate in chromatin remodelling, for example, via the INI-1 complex. However, TACC1 could be an activator in one context and a repressor in another, as shown, for example, for several coregulators such as Zac1 [38]. Although our RNA interference results focus on its role in activation, we cannot exclude the possibility that it plays a role in the repression complex on positive response elements because, in the absence of ligands, TACC1 physically interacts with several NRs including RXR, the common partner to all non-steroid receptors. An interaction between TACCs and RXRβ in the absence of ligand has also been observed [5,25]. TACC1 interactions with both partners on TREs or RAREs could contribute to building the corepressor complex. Heldring *et al*. proposed that N-CoR and SMRT would be recruited to ERα via indirect mechanisms that require additional factors [39]. TACC1 could be one of these factors. It remains to be determined if TACC1 interacts with corepressors or HDACs. Moreover, the siRNAs used were designed to destroy mRNAs of TACC1-A, -K, -S and -J variants, but they could also knock down most mRNA isoforms present in the cells. The effect we observed on TRα1 transcription resulted from the absence of TACC1-A and K proteins, but presumably of many other proteins such as TACC1-S and -J, which the antibody used could not reveal. We cannot exclude the possibility that one of the other isoforms could be a true corepressor. For comparison, N-CoR is a paradigm for corepressors but its natural splice variant N-CoRi is a potent coactivator for unliganded TR [40]. These results, taken as a whole, lead to the suggestion that TACC1 has a dual function and that it is a scaffold protein that organises transcription in NR-complexes. The same conclusion was independently reached by Vettaikkorumakankauv et al [25].

One important observation was that, in TACC1 deprived cells, TRα1 and presumably RARα were delocalized from the nucleus to the perinuclear cytoplasm. This suggests that TACC1 is directly involved in controlling the nuclear localization of both NRs or in regulating the trafficking of these NRs within the chromatin and thereby their availability to target genes. Thus the reduced transcriptional activity mediated by these NRs in TACC1-deprived cells might partly result from this nuclear delocalization. Its importance would depend on the balance between what is displaced in the cytoplasm and what remains in the nucleus. The converse situation is that overexpressed TACC1 can displace endogenous RAR from the nucleus, presumably by interaction in the cytoplasm, as shown by colocalization experiments with TR. Similarly, overexpressed AINT (TACC3) displaced the endogenous nuclear transcription cofactor ARNT [21] and overexpressed TACC1 modulates a transcriptional response of c-fos and c-jun genes [41]. All these results suggest that some TACC1 action on transcription modulation is indirect and takes place from the cytoplasm. It could occur in pathological situations when TACC1 is abnormally present in the cytoplasm or depleted [9-12] in the cell, or under normal situations, as described for TACC3. It maintains the coregulator FOG-1 in the cytoplasm during the undifferentiated state of hematopoietic cells, the terminal differentiation occurring when TACC3 expression is weak, allowing FOG-1 to activate the transcription factor GATA-1 [26].

### TACC1 as a multifunctional protein and a transforming protein

TACC1 appears to be a multifunctional protein similar to many other coregulators [3]. It could work as a scaffold protein organizing different functions around itself. In particular it plays a role in building a structure, the centrosome, and in controlling a function, transcription. A model for such proteins is β-catenin, which forms part of adherens junctions and is a transcription cofactor for TCF/LEF. It has been recently demonstrated that G-actin, a cytoskeleton component when polymerized, regulates gene expression and participates in chromatin remodelling [42]. This sheds light on how the cell can coordinate morphology and transcription. TACC1 might behave in a similar manner, coordinating transcriptional control by several NRs, as well as mitosis.

TACC proteins are involved in several cancers such as mammary, ovarian and gastric cancers, and in leukemia. However their role as oncogenes [7,43] or tumour suppressors [44] has not yet been clarified, as TACC proteins are over- or underexpressed, depending on the tumour cell lines and primary tumours [9-15,45]. Therefore mutations in the structure and function of TACC1 in tumours could mislocalize the protein in a cell subcompartment or deplete one of them. The discovery of TACC interactions with several NRs and previous descriptions of interactions with transcription cofactors has led to the assumption that pathological alterations in the level of expression of TACC1 or in its localization could directly and indirectly affect transcriptional regulation of these NR target genes. For example, it has been described that in the normal human prostate tissue TACC1 is located in the nucleus [31] whereas it is cytoplasmic in prostate adenocarcinomas [11]. It could thereby contribute to oncogenic development, as described for other transcription coregulators [3,46]. It is worth noting that alterations in TACC1 have been found in breast cancers, a type of tumour whose development is known to be controlled by estrogen receptors. The initial description of TACC1 comes from the study of an amplicon associated with ER-positive lobular carcinomas that metastasize to axillary lymph nodes [7]. If amplification in 8p11 is associated with an overexpression of the TACC1 protein, our results suggest that this could perturb the transcription of ER target genes, with consequences for tumorigenesis or the metastasis process. It is interesting that TACC1 has been recognized as the strongest prognostic marker associated with endocrine therapy resistance in breast tumours [45].

## Conclusions

TACC proteins are well-known actors for the mitotic spindle stabilization and the centrosome function [16]. Their role in transcription control is far less documented. Focusing on some Nuclear Hormone Receptors (NRs), we show for the first time that TACC1 plays a role in transcription. The mechanism appears complex as TACC1 behaves as a coactivator since its absence precludes full activation of Thyroid Hormone T3 and retinoic acid-dependent promoters, while the interaction between the studied NRs and TACC1 occurs in the absence of their respective ligands. Overexpression and underexpression have consequences on the subcellular localization of TR and RAR. By its dual function, building the mitotic spindle and regulating transcription, TACC1 might play a role in coordinating the mitosis and transcription. The transforming properties of this protein when misexpressed or mislocalized might be due not only to defects of the spindle but also directly from action on transcription.

## Methods

### Antibodies

The following antibodies were used: mouse monoclonal antibody (mAb) anti-TRα1/β1 (C-1), rabbit polyclonal antibodies anti GR (H-300), ERα(H-20), RXRα(D-20), RARα(C-20), PPARγ(H-100) (Santa Cruz Biotechnologies, Santa Cruz, CA); rabbit polyclonal antibody anti-TACC1 (Upstate cat # 07-229); mAb anti-Flag M2 and mAb anti-Flag M2 HRP (Sigma); anti rabbit and anti mouse IgG Horseradish peroxidase (HRP) conjugate (Promega); anti rabbit and anti mouse Cy3-conjugated IgG and anti rabbit fluorescein (FITC)-conjugated IgG (Jackson) and mouse and rabbit Trueblot (eBioscience).

### Yeast two-hybrid screen

Experiments were performed according to the Clontech Laboratories protocols: manual PT3061-1, for the Matchmaker two-hybrid system 2 in yeast (catalog # K1604-1), and handbook PT3024-1 for the yeast protocols. The full length TRα2 to be used as bait was cloned from a cDNA library built from 17-day mouse embryos (Clontech ML4006AB) to be used as bait. TRα2 is a natural variant form of the thyroid hormone receptor encoded by the TR gene that is unable to bind the ligand T3, in contrast to the receptor TRα1. TRα2 was fused downstream to the GAL4-DBD in pAS2-1. Yeast cells, strains CG1945 and Y190, were transfected in parallel with the plasmid pAS2-1- TRα2 and were selected in the presence of 1 mM or 20 mM 3-AT (aminotriazole), respectively on an SD/-Trp medium. Yeast strains expressing the TRα2-GAL4 DBD were then transfected with a prey cDNA library constructed from 17-day mouse embryo cDNA fused to GAL4-AD in the pGAD10 vector. Transfected clones were selected on an SD/-Trp/-Leu/-His/+3-AT medium (1 mM or 20 mM depending on the strain, see above).

### GST pulldown assay

The GST-TACC1-J fusion construct cloned into the pGEX-3X plasmid were produced in bacteria upon induction with 0.2 mM IPTG at 37°C. The bacteria were sonicated, the lysate was centrifuged and the supernatant incubated with glutathione-Sepharose 4B beads (Pharmacia) for 1 h at 4°C in PBS/0.1 mM PMSF/1% Triton buffer. Beads were washed and protein interactions were performed with [35S]-Met labelled TRα synthesised *in vitro *using TNT kits (Promega). The Sepharose beads were subsequently washed three times with the PBS/PMSF/Triton buffer. Bound proteins were eluted with loading buffer at 100°C and analysed by SDS-PAGE and fluorography (Amplify, Amersham).

### Plasmids and constructs

Human TACC1 cDNAs were amplified from a cDNA library of activated RAJI cells by PCR with the following primers: forward 5'-AGATCTGAATTCCATGGCGTTCAGCCCGTGGC-3', reverse 5'-AGATCTGAATTCTCAGTCAGTCTTTCCCAGC-3'. The four PCR products were subcloned into the pGEM-T vector (Promega, Madison, Wis.) and sequenced. The human TACC1 cDNAs (TACC1-A,-K,-S, and -J) were then cloned into the BglII site of pSG5-Flag vector or pEGFP-C1.

Point mutations (TACC1 amino acids (AA) 609 (Leu) and 610 (Ile) (hTACC1 full-length protein numbering) were introduced using the commercial QuikChange Site-Directed Mutagenesis kit according to the manufacturer's protocol (Stratagene).

The pSG5 rat TRβ1 and pSG5 mouse TRα1 constructs were gifts from ML. Privalsky. The pSG5 rat TRα2 was provided by MA. Lazar. The pGL3 human ERα (hERα), and pSG5 mouse PPARγ constructs were gifts from V. Laudet. pSG5 GR was gift from JM. Vanacker. PSG5 mouse RXRα and RARα were gifts from H. Escriva. The luciferase reporters used in transfection experiments contain chicken E6 lysozyme Thyroid hormone Responsive Element (E6-TRE) from Lee and Privalsky and Retinoic Acid Response Element (RARE) from G. Benoit. Renilla Luciferase used for normalization was from Promega.

Truncated form of TRα1 (TRα1 Δ helix12) was amplified by PCR using High Fidelity polymerase (Roche) and oligonucleotides that introduce EcoRI sites on the 5' and 3' ends, followed by subcloning into the EcoRI site of the pSG5 vector.

### Cell culture

All media were purchased from Invitrogen (Invitrogen/Gibco, Carlsbad, CA). HEK-293F, HeLa and COS-7 cells were maintained in Dulbecco's Modified Eagle's Medium (DMEM) supplemented with 6% heat-inactivated fetal bovine serum, glutamine (2 mM), penicillin/streptomycin (1000 U/ml). F9 mouse embryonic carcinoma cells were cultured on 0.1% gelatin-coated plates in the medium described above but supplemented with MEM sodium pyruvate (1 mM). Cells were maintained at 37°C in the presence of 5% CO_2_. Whenever indicated, charcoal-stripped fetal bovine Serum (Biochrom, AG) was used and hormones were used at 10^-6^M or 10^-7^M.

### Indirect immunofluorescence microscopy

Cells were seeded overnight on coverslips onto a 24 -well plate and transfected or not with 0.5-1 μg of DNA with Exgen500 (Fermentas protocol). 48 h after transfection, cells were fixed for 15 min in 0.5% formaldehyde/1.5% glutaraldehyde diluted in PBS. After extensive washing, cells were subsequently overlaid with the appropriate antibody (Santa Cruz Biotechnologies, Santa Cruz, CA) diluted in 5% normal goat serum in the presence of 0.1% Triton X-100 for 1 h at room temperature. After washing, anti-mouse or rabbit Cy3 or FITC conjugated secondary antibody (Jackson ImmunoResearch Laboratories) was added and the cell nuclei were later counterstained with DAPI (4', 6-diamidino-2-phenylindole dihydrochloride hydrate) (Sigma), Propidium Iodide or DRAQ5 (Biostatus limited). The coverslips were mounted on microscopy slides with FluorSave embedding medium (Calbiochem) and imaged on the appropriate fluorescence microscope.

### Co-immunoprecipitation

Cos-7 cells were transfected using lipofectamin according to the manufacturer's protocol (Invitrogen/Gibco, Carlsbad, CA). Cotransfected cell lysates were pre-cleared at 4°C for 2 h by adding 100 μl protein G-Sepharose bead slurry (50%) per ml (Amersham) or anti-Flag M2 affinity gel (Sigma), and 800 μg were incubated at 4 C for 2 h in the presence of the appropriate antibody (2 μg) or of a non specific control antibody (2 μg). The lysates were then incubated overnight at 4°C with 100 μl protein G-Sepharose (50% slurry) (Amersham) or anti-Flag M2 affinity gel (Sigma). The Sepharose was washed twice with RIPA buffer and three times with the washing buffer (20 mM Tris pH 8, 150 mM NaCl, 2 mM EDTA, 1% Triton ×100) and immobilized proteins were eluted with 50 μl protein gel loading buffer. Immunoprecipitation was followed by Western blotting to detect bound proteins.

### Western blot analysis

Proteins were separated by SDS-PAGE then transferred to nitrocellulose membranes, which were incubated for 2 hours at room temperature with blocking buffer [TBST (20 mM Tris-HCl pH 7.6, 150 mM NaCl, 0.1% Tween 20) + 2% non-fat dry milk]. After extensive washing with TBST, the blots were hybridized for 1 hour with primary antibodies followed by 1 hour incubation at room temperature with HRP-conjugated secondary antibodies against mouse or rabbit IgG (Promega, Madison, Wis.) or true-blot detection system (eBioscience) accordingly. The proteins were detected using SuperSignal^® ^West Pico Chemiluminescent, as recommended by the manufacturer (Pierce, Perbio) and autoradiographed.

### Subcellular fractionations

Total proteins were extracted 48 h after transfection in RIPA buffer (50 mM Tris pH 8, 140 mM NaCl, 1 mM EDTA, 0.5 mM EGTA, 1% Triton ×100, 0.1% Na-deoxycholate) supplemented with Protease Inhibitor Cocktail (Complete, Roche). After homogenisation at 4°C for 20 min, samples were centrifuged at 10,000 × g for 15 min at 4°C and the supernatant containing proteins frozen and stored at -80°C. Nuclear and cytoplasmic proteins were isolated using Chemicon's Nuclear Extraction Kit according to the manufacturer's protocol.

For F9 subcellular fractionation, cells were collected in ice cold 1 × PBS and re-suspended at 4 × 10^7 ^cells/ml in buffer A (10 mM HEPES pH 7.9, 10 mM KCl, 1.5 mM MgCl_2_, 0.34 M sucrose, 10% glycerol, 1 mM dithiothreitol, and protease inhibitor cocktail [Roche]). Triton X-100 was added (0.1% final concentration), the cells were incubated on ice for 8 min, and the nuclei (fraction P1) were collected by centrifugation (5 min, 1,300 × g, 4°C). The supernatant (fraction S1) was clarified by high-speed centrifugation (5 min, 20,000 × g, 4°C), and the supernatant (fraction S2) collected. The P1 nuclei were washed once in buffer A and lysed for 30 min in buffer B [3 mM EDTA, 0.2 mM EGTA, 1 mM dithiothreitol and protease inhibitor cocktail (Roche)]. Insoluble chromatin (fraction P3) and soluble (fraction S3) fractions were separated by centrifugation (5 min, 1,700 × g, 4°C). The P3 fraction was washed once with buffer B and re-suspended in SDS-Laemmli buffer and boiled for 10 min. Protein concentrations were determined by BCA protein assay (Interchim).

### RNA interference

Synthetic double-strand siRNA specific for the human TACC1-J coding region (Custom Smart Pool siRNA) and the siRNA ON-TARGET plus siCONTROL non-targeting siRNA n°1 were purchased from Dharmacon (Perbio). The day before transfection, COS-7 and HEK-293F cells were seeded at 5 × 10^4 ^and 1.5 × 10^5 ^cells/ml, respectively. Cells were transfected with 50 nM of siRNA, using Dharmafect 1 (Dharmacon, Perbio) according to the manufacturer's protocol.

### Luciferase assay

For transactivation-reporter assays, cells were seeded onto 24-well plates overnight and transfected with DNA and Exgen500 according to the manufacturer's protocol (Fermentas). Transfected cells in each well were lysed and processed for a dual luciferase assay according to the manufacturer's protocol (Dual-Luciferase Reporter Assay, Promega, Madison, Wis.). Luminescence was determined with a luminometer (Veritas, Microplate Luminometer, Turner Biosystems). Relative luciferase activity (RLU) was calculated as the Firefly luciferase activity normalized to Renilla luciferase activity from cotransfected pRL-CMV-luc.

### Quantitative RT-PCR

For quantitative analysis real-time quantitative PCR was carried out using primers designed with the Primer 3 software and produced by MWG (see Additional file 5). The Mx3000p Detection system (Stratagene) was used with QuantiTect SYBR Green PCR Master Mix (Qiagen) for the detection of PCR products. The cycle threshold was set at a level where the exponential increase in PCR amplification was approximately equal between all samples.

## Abbreviations

TACC: transforming acidic coiled coil; NR: nuclear receptor; TR: thyroid hormone receptor; RAR: retinoic acid receptor; RXR: retinoic × receptor; ER: estrogen receptor; GR: glucocorticoid receptor; PPAR: peroxisome proliferator-activated receptor; AR: androgen receptor; ATRA: all-trans retinoic acid; T3: triiodothyronin; CC domain: coiled-coil domain; AA: amino acid.

## Authors' contributions

RG performed many of the experiments described in this article: co-immunoprecipitations, subcellular fractionations and interactions, part of siRNA experiments, immunofluorescence and confocal experiments; he participated in experiment design and helped to draft the manuscript. SV carried out the colocalization experiments in HeLa and COS-7 cells, observed the decreased interaction in yeast cells in the presence of T3, and defined in yeast the interaction domains in TACC1 and TR. JB performed part of the siRNA experiments. JS, the team leader, conceived of the study and participated in its design and coordination. PRC performed the yeast two-hybrid experiments, the GST-pulldown assay, the functional interactions in HeLa cells, supervised the JB' work, conceived of the study and participated in its design and coordination and drafted the manuscript. All authors read and approved the final manuscript.

## Supplementary Material

Additional file 1 **TACC1 interacts with TRα1 and TRα2 in yeast**. The interaction between TACC1-Y11 and TRα1 or TRα2 was analysed in a yeast two-hybrid assay, in the absence or presence of the thyroid hormone [T3 (10^-7^M)]. Interaction was quantified by measuring the β-gal units produced by the activated reporter. Click here for file

Additional file 2 **TACC1 interacts with both steroid and non- steroid nuclear receptors**. The Flag-TACC1-J expression plasmid was cotransfected with RARα (A), PPARγ(B), ERα(C), RXRα(D) or GR(E) expression plasmids into COS-7 cells in the absence or presence of their respective ligands at the indicated concentrations. 48 h after transfection, whole cell lysates were prepared and immunoprecipitated with anti-RAR, -RXR, -PPAR, -ER or -GR antibody. Immunoprecipitates were resolved by SDS-PAGE and blotted with anti-flag antibody. Inputs correspond to 1% of the proteins used for the coimmunoprecipitation assay. Click here for file

Additional file 3 **Interaction between overexpressed TACC1 and endogenous RARα**. Cos7 cells were transfected (C), or not, with GFP TACC1-A and immunofluoresence was performed against endogenous RARα(D, E). We observed the colocalization of overexpressed TACC1 with endogenous RARα (G). It appeared also that overexpressed TACC1 delocalized RARα from the nucleus to the cytoplasm; in non transfected cells RARα is nuclear (F), whereas it is mainly cytoplasmic in transfected cells (G). Note that overexpressed TACC1 was mainly cytoplasmic, surrounding the nucleus, forming a structure that certainly corresponds to aggregates previously described by Gergely and collaborators [47]. A and B are DAPI stainings. Click here for file

Additional file 4 **Decrease of TACC1 mRNA level using specific targeted TACC1 siRNA**. The efficiency of TACC1 siRNA (50 nM) versus Non Targeting siRNA (NT) was verified by RT-PCR analysis in Cos-7 and HEK-293F cells. All TACC1 isoforms were amplified and HPRT was used as internal control gene (A). TACC1 siRNA specificity was verified by amplification of TACC1 (white), TACC2 (grey) and TACC3 (black) by quantitative RT-PCR in Cos-7 and HEK-293F cells. The results were normalised with 36B4 as a control mRNA (B). Click here for file

Additional file 5 **A table of oligonucleotides**. Oligonucleotides used in the experiments. Click here for file

## References

[B1] Jepsen K Rosenfeld MG Biological roles and mechanistic actions of co-repressor complexes Journal of cell science 2002 115 Pt 4 689 698 11865025 10.1242/jcs.115.4.689

[B2] Rosenfeld MG Lunyak VV Glass CK Sensors and signals: a coactivator/corepressor/epigenetic code for integrating signal-dependent programs of transcriptional response Genes & development 2006 20 11 1405 1428 10.1101/gad.1424806 16751179

[B3] Lonard DM Lanz RB O'Malley BW Nuclear receptor coregulators and human disease Endocrine reviews 2007 28 5 575 587 10.1210/er.2007-0012 17609497

[B4] Lonard DM O'Malley BW Expanding functional diversity of the coactivators Trends in biochemical sciences 2005 30 3 126 132 10.1016/j.tibs.2005.01.001 15752984

[B5] Still IH Vettaikkorumakankauv AK DiMatteo A Liang P Structure-function evolution of the transforming acidic coiled coil genes revealed by analysis of phylogenetically diverse organisms BMC Evol Biol 2004 4 16 15207008 10.1186/1471-2148-4-16 PMC441373

[B6] Stewart JP Thompson A Santra M Barlogie B Lappin TR Shaughnessy J Jr Correlation of TACC3, FGFR3, MMSET and p21 expression with the t(4;14)(p16.3;q32) in multiple myeloma British journal of haematology 2004 126 1 72 76 10.1111/j.1365-2141.2004.04996.x 15198734

[B7] Still IH Hamilton M Vince P Wolfman A Cowell JK Cloning of TACC1, an embryonicallyexpressed potentially transforming coiled coil containing gene from the 8p11 breast cancer amplicon Oncogene 1999 18 27 4032 4038 10.1038/sj.onc.1202801 10435627

[B8] Still IH Vince P Cowell JK The third member of the transforming acidic coiled coil-containing gene family TACC3, maps in 4p16, close to translocation breakpoints in multiplemyeloma and is upregulated in various cancer cell lines Genomics 1999 58 2 165 170 10.1006/geno.1999.5829 10366448

[B9] Conte N Charafe-Jauffret E Delaval B Adelaide J Ginestier C Geneix J Isnardon D Jacquemier J Birnbaum D Carcinogenesis and translational controls: TACC1 is down-regulated in human cancers and associates with mRNA regulators Oncogene 2002 21 36 5619 5630 10.1038/sj.onc.1205658 12165861

[B10] Conte N Delaval B Ginestier C Ferrand A Isnardon D Larroque C Prigent C Seraphin B Jacquemier J Birnbaum D TACC1-chTOG-Aurora A protein complex in breast cancer Oncogene 2003 22 50 8102 8116 10.1038/sj.onc.1206972 14603251

[B11] Devilard E Bladou F Ramuz O Karsenty G Dales JP Gravis G Nguyen C Bertucci F Xerri L Birnbaum D FGFR1 and WT1 are markers of human prostate cancer progression BMC Cancer 2006 6 272 10.1186/1471-2407-6-272 17137506 PMC1698935

[B12] Lauffart B Vaughan MM Eddy R Chervinsky D DiCioccio RA Black JD Still IH Aberrations of TACC1 and TACC3 are associated with ovarian cancer BMC Womens Health 2005 5 8 10.1186/1472-6874-5-8 15918899 PMC1175095

[B13] Line A Slucka Z Stengrevics A Silina K Li G Rees RC Characterisation of tumour-associated antigens in colon cancer Cancer Immunol Immunother 2002 51 10 574 582 10.1007/s00262-002-0322-2 12384809 PMC11032767

[B14] Line A Stengrevics A Slucka Z Li G Jankevics E Rees RC Serological identification and expression analysis of gastric cancer-associated genes British journal of cancer 2002 86 11 1824 1830 10.1038/sj.bjc.6600321 12087473 PMC2375403

[B15] Partheen K Levan K Osterberg L Horvath G Expression analysis of stage III serous ovarian adenocarcinoma distinguishes a sub-group of survivors Eur J Cancer 2006 42 16 2846 2854 10.1016/j.ejca.2006.06.026 16996261

[B16] Gergely F Centrosomal TACCtics Bioessays 2002 24 10 915 925 10.1002/bies.10162 12325124

[B17] Theurkauf WE TACCing down the spindle poles Nature cell biology 2001 3 7 E159 161 10.1038/35083110 11433309

[B18] Peset I Vernos I The TACC proteins: TACC-ling microtubule dynamics and centrosome function Trends in cell biology 2008 18 8 379 388 10.1016/j.tcb.2008.06.005 18656360

[B19] Raff JW Centrosomes and cancer: lessons from a TACC Trends in cell biology 2002 12 5 222 225 10.1016/S0962-8924(02)02268-7 12062169

[B20] Stebbins-Boaz B Cao Q de Moor CH Mendez R Richter JD Maskin is a CPEB-associated factor that transiently interacts with elF-4E Molecular cell 1999 4 6 1017 1027 10.1016/S1097-2765(00)80230-0 10635326

[B21] Sadek CM Jalaguier S Feeney EP Aitola M Damdimopoulos AE Pelto-Huikko M Gustafsson JA Isolation and characterization of AINT: a novel ARNT interacting protein expressed during murine embryonic development Mechanisms of development 2000 97 1-2 13 26 10.1016/S0925-4773(00)00415-9 11025203

[B22] Gangisetty O Lauffart B Sondarva GV Chelsea DM Still IH The transforming acidic coiled coil proteins interact with nuclear histone acetyltransferases Oncogene 2004 23 14 2559 2563 10.1038/sj.onc.1207424 14767476

[B23] Lauffart B Gangisetty O Still IH Molecular cloning genomic structure and interactions of the putative breast tumor suppressor TACC2 Genomics 2003 81 2 192 201 10.1016/S0888-7543(02)00039-3 12620397

[B24] Lauffart B Howell SJ Tasch JE Cowell JK Still IH Interaction of the transforming acidic coiled-coil 1 (TACC1) protein with ch-TOG and GAS41/NuBI1 suggests multiple TACC1-containing protein complexes in human cells Biochem J 2002 363 Pt 1 195 200 10.1042/0264-6021:3630195 11903063 PMC1222467

[B25] Vettaikkorumakankauv AKLB Gangisetty O Cincotta MA Hawthorne LA Cowell JK Still IH The TACC proteins are coregulators of the rentinoid × Receptor β Cancer Therapy 2008 6 805 816

[B26] Garriga-Canut M Orkin SH Transforming acidic coiled-coil protein 3 (TACC3) controls friend of GATA-1 (FOG-1) subcellular localization and regulates the association between GATA-1 and FOG-1 during hematopoiesis The Journal of biological chemistry 2004 279 22 23597 23605 10.1074/jbc.M313987200 15037632

[B27] Angrisano T Lembo F Pero R Natale F Fusco A Avvedimento VE Bruni CB Chiariotti L TACC3 mediates the association of MBD2 with histone acetyltransferases and relieves transcriptional repression of methylated promoters Nucleic acids research 2006 34 1 364 372 10.1093/nar/gkj400 16410616 PMC1331987

[B28] Tagami T Kopp P Johnson W Arseven OK Jameson JL The thyroid hormone receptor variant alpha2 is a weak antagonist because it is deficient in interactions with nuclear receptor corepressors Endocrinology 1998 139 5 2535 2544 10.1210/en.139.5.2535 9564869

[B29] Selmi-Ruby S Casanova J Malhotra S Roussett B Raaka BM Samuels HH Role of the conserved C-terminal region of thyroid hormone receptor-alpha in ligand-dependent transcriptional activation Molecular and cellular endocrinology 1998 138 1-2 105 114 10.1016/S0303-7207(98)00016-1 9685219

[B30] Line A Slucka Z Stengrevics A Li G Rees RC Altered splicing pattern of TACC1 mRNA in gastric cancer Cancer genetics and cytogenetics 2002 139 1 78 83 10.1016/S0165-4608(02)00607-6 12547166

[B31] Lauffart B Dimatteo A Vaughan MM Cincotta MA Black JD Still IH Temporal and spatial expression of TACC1 in the mouse and human Dev Dyn 2006 235 6 1638 1647 10.1002/dvdy.20724 16496324

[B32] Vasios G Mader S Gold JD Leid M Lutz Y Gaub MP Chambon P Gudas L The late retinoic acid induction of laminin B1 gene transcription involves RAR binding to the responsive element The EMBO journal 1991 10 5 1149 1158 1850696 10.1002/j.1460-2075.1991.tb08055.x PMC452768

[B33] de The H Vivanco-Ruiz MM Tiollais P Stunnenberg H Dejean A Identification of a retinoic acid responsive element in the retinoic acid receptor beta gene Nature 1990 343 6254 177 180 10.1038/343177a0 2153268

[B34] Langston AW Gudas LJ Identification of a retinoic acid responsive enhancer 3' of the murine homeobox gene Hox-1.6 Mechanisms of development 1992 38 3 217 227 10.1016/0925-4773(92)90055-O 1360810

[B35] Gergely F Draviam VM Raff JW The ch-TOG/XMAP215 protein is essential for spindle pole organization in human somatic cells Genes & development 2003 17 3 336 341 10.1101/gad.245603 PMC195983 12569123

[B36] Anafi M Yang YF Barlev NA Govindan MV Berger SL Butt TR Walfish PG GCN5 and ADA adaptor proteins regulate triiodothyronine/GRIP1 and SRC-1 coactivator-dependent gene activation by the human thyroid hormone receptor Molecular endocrinology (Baltimore, Md) 2000 14 5 718 732 10.1210/me.14.5.718 10809234

[B37] Robyr D Wolffe AP Wahli W Nuclear hormone receptor coregulators in action: diversity for shared tasks Molecular endocrinology (Baltimore, Md) 2000 14 3 329 347 10.1210/me.14.3.329 10707952

[B38] Huang SM Stallcup MR Mouse Zac1, a transcriptional coactivator and repressor for nuclear receptors Molecular and cellular biology 2000 20 5 1855 1867 10.1128/MCB.20.5.1855-1867.2000 10669760 PMC85366

[B39] Heldring N Pawson T McDonnell D Treuter E Gustafsson JA Pike AC Structural insights into corepressor recognition by antagonist-bound estrogen receptors The Journal of biological chemistry 2007 282 14 10449 10455 10.1074/jbc.M611424200 17283072

[B40] Meng X Webb P Yang YF Shuen M Yousef AF Baxter JD Mymryk JS Walfish PG E1A and a nuclear receptor corepressor splice variant (N-CoRI) are thyroid hormone receptor coactivators that bind in the corepressor mode Proceedings of the National Academy of Sciences of the United States of America 2005 102 18 6267 6272 10.1073/pnas.0501491102 15849266 PMC1088377

[B41] Lauffart B Sondarva GV Gangisetty O Cincotta M Still IH Interaction of TACC proteins with the FHL family: implications for ERK signaling Journal of cell communication and signaling 2007 1 1 5 15 10.1007/s12079-007-0001-3 18481206 PMC2267652

[B42] Vartiainen MK Guettler S Larijani B Treisman R Nuclear actin regulates dynamic subcellular localization and activity of the SRF cofactor MAL Science (New York NY) 2007 316 5832 1749 1752 10.1126/science.1141084 17588931

[B43] Cully M Shiu J Piekorz RP Muller WJ Done SJ Mak TW Transforming acidic coiled coil 1 promotes transformation and mammary tumorigenesis Cancer research 2005 65 22 10363 10370 10.1158/0008-5472.CAN-05-1633 16288026

[B44] Chen HM Schmeichel KL Mian IS Lelievre S Petersen OW Bissell MJ AZU-1: a candidate breast tumor suppressor and biomarker for tumor progression Mol Biol Cell 2000 11 4 1357 1367 10749935 10.1091/mbc.11.4.1357 PMC14852

[B45] Ghayad SE Vendrell JA Bieche I Spyratos F Dumontet C Treilleux I Lidereau R Cohen PA Identification of TACC1, NOV, and PTTG1 as new candidate genes associated with endocrine therapy resistance in breast cancer Journal of molecular endocrinology 2009 42 2 87 103 10.1677/JME-08-0076 18984771

[B46] Bonamy GM Allison LA Oncogenic conversion of the thyroid hormone receptor by altered nuclear transport Nuclear receptor signaling 2006 4 e008 16741566 10.1621/nrs.04008 PMC1472669

[B47] Gergely F Karlsson C Still I Cowell J Kilmartin J Raff JW The TACC domain identifies a family of centrosomal proteins that can interact with microtubules Proc Natl Acad Sci USA 2000 97 26 14352 14357 10.1073/pnas.97.26.14352 11121038 PMC18922

