# Supplementary figures and images for: The transforming acidic coiled coil (TACC1) protein modulates the transcriptional activity of the nuclear receptors TR and RAR
Source: BMC Mol Biol. 2010 Jan 15;11:3. doi: 10.1186/1471-2199-11-3 (PMC2822774; doi:10.1186/1471-2199-11-3)

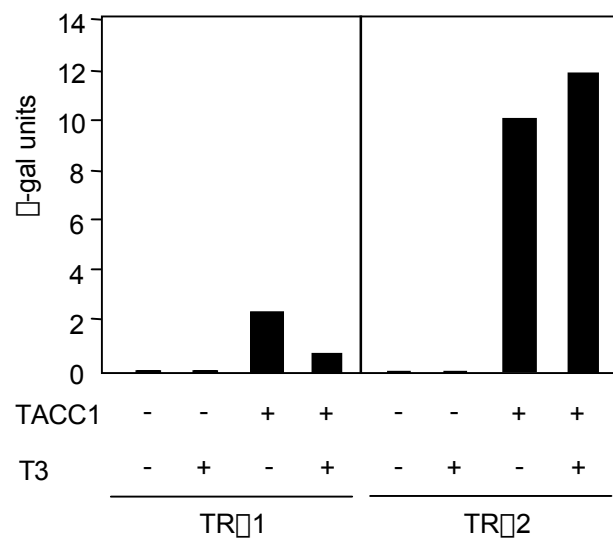

Supplement: Additional file 1 — TACC1 interacts with TRα1 and TRα2 in yeast. The interaction between TACC1-Y11 and TRα1 or TRα2 was analysed in a yeast two-hybrid assay, in the absence or presence of the thyroid hormone [T3 (10-7M)]. Interaction was quantified by measuring the β-gal units produced by the activated reporter. [file 1471-2199-11-3-S1.PDF]

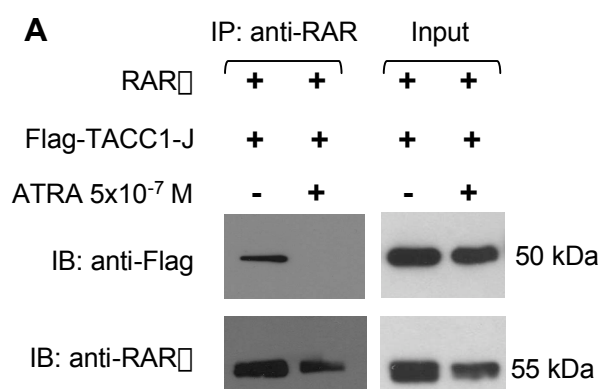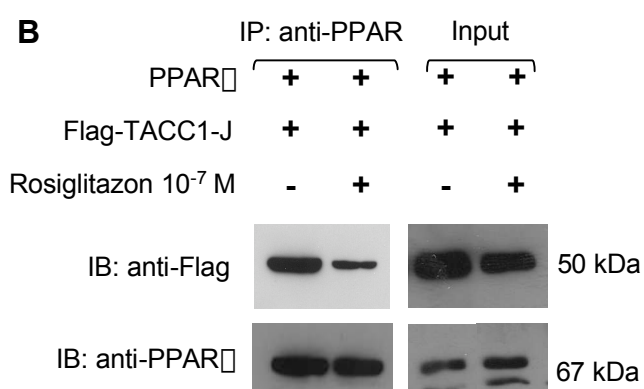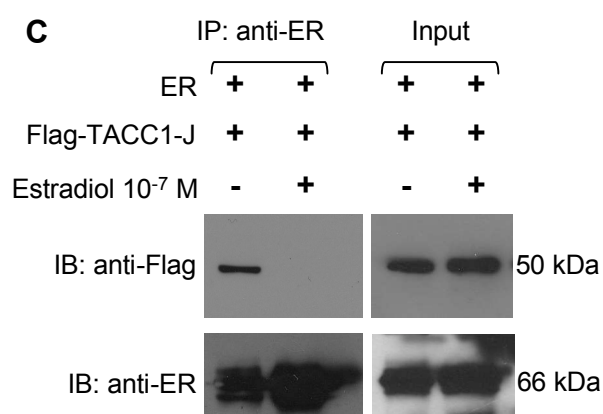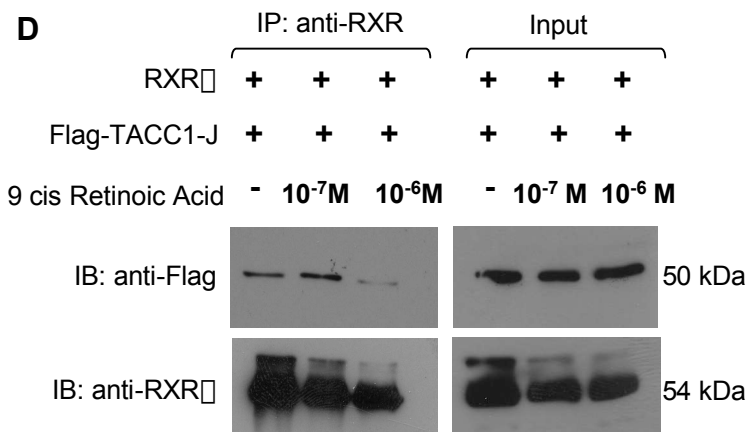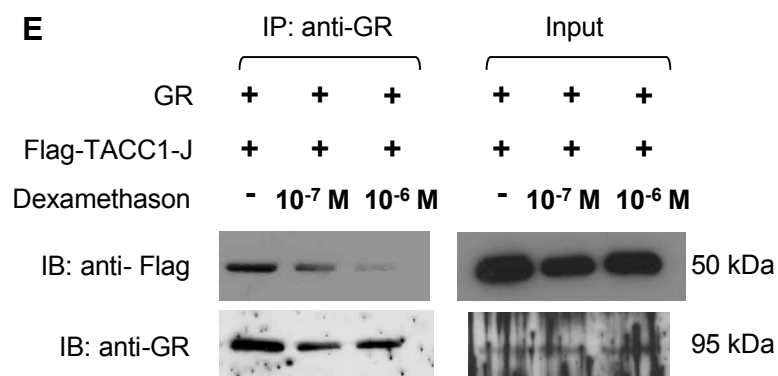

Supplement: Additional file 2 — TACC1 interacts with both steroid and non- steroid nuclear receptors. The Flag-TACC1-J expression plasmid was cotransfected with RARα (A), PPARγ(B), ERα(C), RXRα(D) or GR(E) expression plasmids into COS-7 cells in the absence or presence of their respective ligands at the indicated concentrations. 48 h after transfection, whole cell lysates were prepared and immunoprecipitated with anti-RAR, -RXR, -PPAR, -ER or -GR antibody. Immunoprecipitates were resolved by SDS-PAGE and blotted with anti-flag antibody. Inputs correspond to 1% of the proteins used for the coimmunoprecipitation assay. [file 1471-2199-11-3-S2.PDF]

DAPI

GFP-TACC1-A

RAR $\alpha$

merge

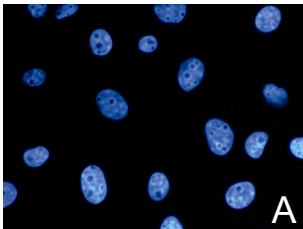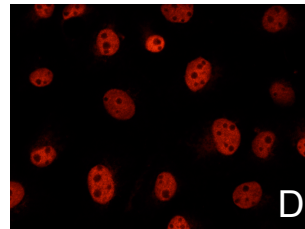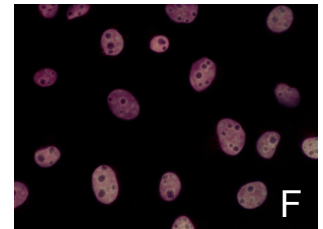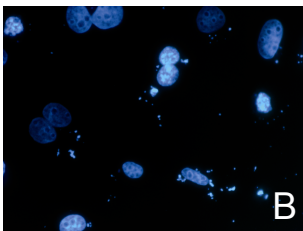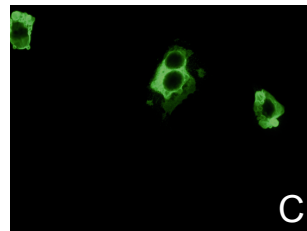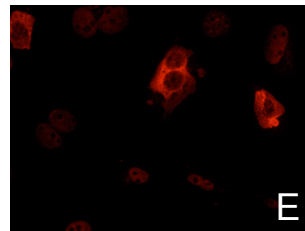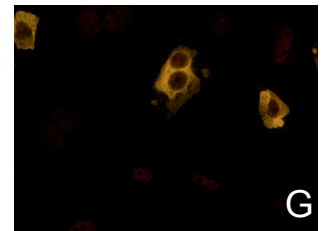

Supplement: Additional file 3 — Interaction between overexpressed TACC1 and endogenous RARα. Cos7 cells were transfected (C), or not, with GFP TACC1-A and immunofluoresence was performed against endogenous RARα(D, E). We observed the colocalization of overexpressed TACC1 with endogenous RARα (G). It appeared also that overexpressed TACC1 delocalized RARα from the nucleus to the cytoplasm; in non transfected cells RARα is nuclear (F), whereas it is mainly cytoplasmic in transfected cells (G). Note that overexpressed TACC1 was mainly cytoplasmic, surrounding the nucleus, forming a structure that certainly corresponds to aggregates previously described by Gergely and collaborators [47]. A and B are DAPI stainings. [file 1471-2199-11-3-S3.PDF]

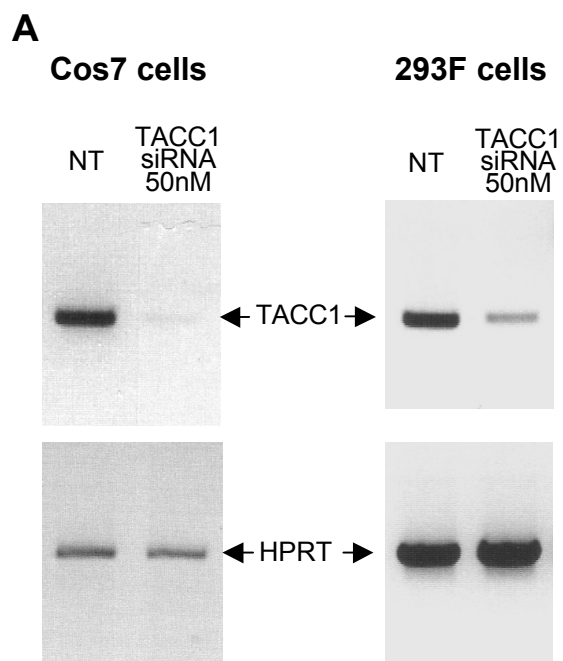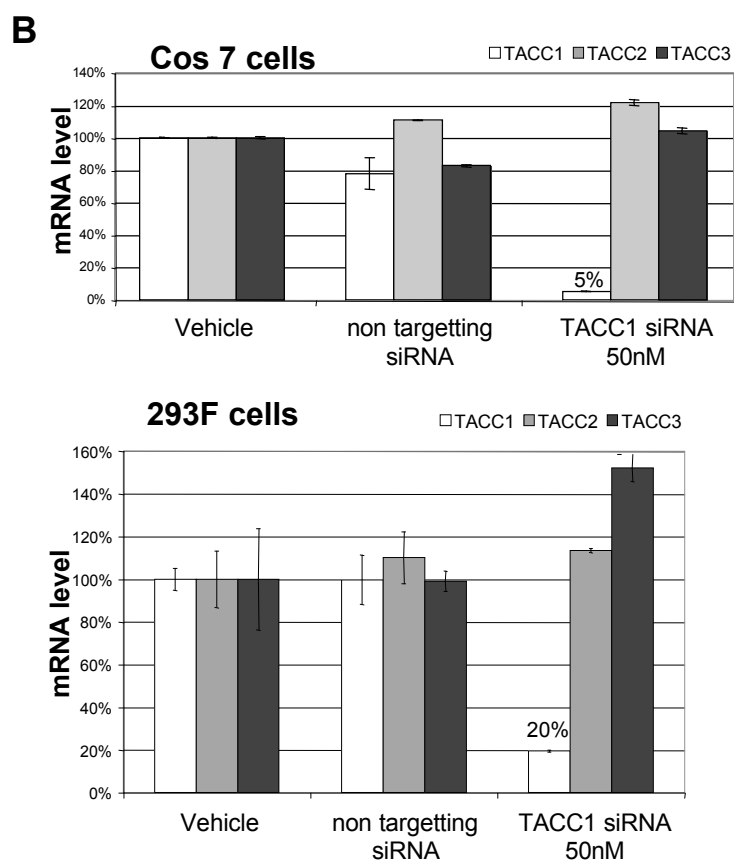

Supplement: Additional file 4 — Decrease of TACC1 mRNA level using specific targeted TACC1 siRNA. The efficiency of TACC1 siRNA (50 nM) versus Non Targeting siRNA (NT) was verified by RT-PCR analysis in Cos-7 and HEK-293F cells. All TACC1 isoforms were amplified and HPRT was used as internal control gene (A). TACC1 siRNA specificity was verified by amplification of TACC1 (white), TACC2 (grey) and TACC3 (black) by quantitative RT-PCR in Cos-7 and HEK-293F cells. The results were normalised with 36B4 as a control mRNA (B). [file 1471-2199-11-3-S4.PDF]
