# Supplementary material for: The transforming acidic coiled coil (TACC1) protein modulates the transcriptional activity of the nuclear receptors TR and RAR
Source: BMC Mol Biol. 2010 Jan 15;11:3. doi: 10.1186/1471-2199-11-3 (PMC2822774; doi:10.1186/1471-2199-11-3)
Supplement: Additional file 5 — A table of oligonucleotides. Oligonucleotides used in the experiments. [file 1471-2199-11-3-S5.PDF]

Gene	Sequence	Tm (°C)
hTACC1long-F	5'-GCAGGAAACTGGGTAGCA-3'	56.0
hTACC1long-R	5'-GGCTTGGAAGATGTTTGG-3'	53.7
hTACC1all-F	5'-ACAGCCCTGGAAGAAACC-3'	56.0
hTACC1all-R	5'-TCAGCAGCCTAAGGGAAC-3'	56.0
hTACC2-F	5'-TAAATCCCCAGCCTCCTT-3'	53.7
hTACC2-R	5'-GGGGCGTCTTCTTCTTCT-3'	56.0
hTACC3-F	5'-TTCTTGGGAGCACTGGAC-3'	56.0
hTACC3-R	5'-TCCTGTGTCGCCTTTACC-3'	56.0
hRAR□4-F	5'-TGAGGAACTCGTCCCAAG-3'	56.0
hRAR□4-R	5'-GCCCTTACATCCCTCACA-3'	56.0
hLaminin B1-F	5'-TGACACGACAGACCCAGA-3'	56.0
hLaminin B1-R	5'-TATCCAAACCGGCAGAAC-3'	53.7
hHOXa1-F	5'-AACCTGGGGGTGTCCTAC-3'	58.2
hHOXa1-R	5'-TTCTTGGTGGGTCTGCTT-3'	53.7
h36B4-F	5'-AACTCTGCATTCTCGCTTCCT-3'	57.9
h36B4-R	5'-ACTCGTTTGTACCCGTTGATG-3'	57.9
